# Disabled People or Their Support Persons’ Perceptions of a Community Based Multi-Sensory Environment (MSE): A Mixed-Method Study

**DOI:** 10.3390/ijerph20196805

**Published:** 2023-09-22

**Authors:** Amanda Wilkinson, Allyson Calder, Beth Elliott, Ryan Rodger, Hilda Mulligan, Leigh Hale, Meredith Perry

**Affiliations:** 1 Centre for Health, Activity and Rehabilitation Research, School of Physiotherapy, University of Otago, Dunedin 9054, New Zealand; mandy.wilkinson@otago.ac.nz (A.W.); ally.calder@otago.ac.nz (A.C.); bethelliott676@hotmail.com (B.E.); ryan.rodger@ccdhb.org.nz (R.R.); leigh.hale@otago.ac.nz (L.H.); 2 Canterbury Multi-Sensory Trust, Christchurch 8024, New Zealand; hilda.mulligan@gmail.com

**Keywords:** multi-sensory environments, disabled people, accessibility, community-based facilities, social connection, culturally safe, self-determination, autonomy, choice, control

## Abstract

Multi-sensory environments (MSEs) are specialised spaces purposely designed to stimulate the senses, whilst providing a calming and relaxing environment for leisure and enjoyment, predominantly intended for disabled people. Most MSEs are in institutions, hospitals, or educational settings, with a few in community-based settings. We explored disabled users’ experiences of a community based MSE in a large metropolitan area in New Zealand, with a view to expanding access to MSE-type environments within the area. We used a convergent mixed method design with a web-based electronic survey (e-survey; *n* = 105), as well as semi-structured interviews (*n* = 14) with disabled MSE users (adults and children), who were supported, where necessary, by their support person/s. We collected the MSE users’ demographics, frequency of use with respect to age, disability, and ethnicity, and experiences of the room, equipment, and accessibility. The participants and their support persons’ perspectives about their experiences of using the MSE were represented by four themes: (i) Self-determination; (ii) Enhancing wellbeing opportunities; (iii) the MSE itself; (iv) Accessibility. While the MSE was considered positively, the MSE experience could be enhanced by addressing access challenges and broadening the scope of equipment to improve the usability and make it a more inclusive environment for all.

## 1. Introduction

Multi-sensory environments (MSEs) are rooms or specialised spaces purposely designed to stimulate all of the senses, whilst providing a calming and relaxing environment for leisure and enjoyment, predominantly for disabled people [1,2,3]. MSE spaces are also used for education, therapeutic, and interactive purposes [4] because they are associated with social, psychological, behavioural, and physical effects [1,5,6,7,8]. MSEs were developed as leisure activities for people with intellectual and learning needs and people with complex needs, as defined by Dutch therapists, Ad Verheul and Jan Hulsegge [3,5,9]. Such environments are now described as ‘comfort rooms’ [10] and ‘sensory rooms’ [11]. Although their names vary, the rooms have common features, including the ability to block out natural light, padded floors, equipment, and walls that ensure room users can explore safely [3]. To aid therapy, education, and relaxation, the stimulation can and should be tailored to individual’s needs with an element of room user control [6,10]. MSEs are now used with a range of disabled populations, including people with dementia, autism spectrum disorder (ASD), sensory processing disorders, and intellectual and developmental disabilities (IDD) [2,4,7]. Most MSEs are located in institutions, hospitals, or educational settings [2,8], with very few in community-based settings. At present, there are only a few community-based MSEs in New Zealand and, to the best of our knowledge, no research has been undertaken pertinent to this country.

One community-based MSE, situated in a large metropolitan centre in NZ, was developed about 20 years ago, and was specifically developed in order to enhance community recreation and leisure opportunities for disabled people. The MSE is community owned through a partnership agreement between the City Council’s Leisure Unit and a Charitable Trust. Located in suburbia, the MSE is upstairs in a community-based facility containing indoor swimming pools, a gym and fitness centre, and a stadium with basketball, netball, volleyball, and badminton courts. The MSE room has a variety of equipment such as sound, various forms of lighting, interactive activities/toys, soft and hard furniture for sitting or lying, and a ‘smelling’ box. The MSE room combines aspects of the “Snoezelen” approach (a structured approach) with an interactive component to enable people to interact with the room passively or actively, and offers the freedom to control, manipulate, intensify, reduce, or isolate the sensory stimulation. It is open during office hours (9–4.30 pm) in the week and for four hours on each day of the weekend. It can be booked for use by individuals or groups of children or adults, and even, for example, for children’s birthday parties, irrespective of whether the users are disabled or not.

Disabled people are a large, diverse minority group and an important part of every society [12]. Yet this group face challenges in exercising their right to full and effective participation in community recreation and leisure activities [13,14,15,16,17]. Disability is not due to the individual’s impairments, but due to social and environmental factors that impact people and create barriers to full participation (see Social Model of Disability) [18,19]. Active participation in community-based leisure and recreational activities contributes to both able and disabled people’s health and well-being [20]. It is important that community spaces are therefore accessible and are designed to ensure that all feel valued, included, and can actively participate in their community [16,18]. To date, there have been very few clinical trials supporting the effectiveness of MSEs, and the existing research is limited and inconsistent [2]. Typically, staff perceptions of therapy sessions (held in rest homes, hospitals, and special education schools) suggest decreased aggression and disruptive behaviours have been observed in, for example, people with intellectual and developmental disabilities [7] and dementia [21]. It appears that there is little known about what disabled people think of MSEs.

The City Council and Charitable Trust invited the research team to explore disabled users’ experiences of the MSE that they operate and support with a view to expanding access to MSE-type environments within the metropolitan area. Additionally, given the paucity of evidence internationally and nationally of the benefits of community-based MSEs, it was deemed relevant to understand who uses the MSE and their perceptions of it.

## 2. Materials and Methods

Aspects of interest in this study were MSE user demographics, frequency of MSE use with respect to age, disability, and ethnicity, and participants’ experiences of the room, equipment, and accessibility of this one specific MSE operated by the collaborating City Council. A convergent mixed method design [22] was deemed the best way to meet the study objectives. The study used a web-based electronic survey (e-survey) and a qualitative descriptive study using semi-structured interviews with disabled MSE users (adults and children), supported, where necessary, by their carers or support person/s. The University of Otago Ethics Committee (Health) (Ref H22/040) provided ethical approval for the study following consultation with the Ngāi Tahu Research Committee, University of Otago, and locality agreement from the Recreation and Sport Services Managers of the relevant City Council. The Checklist for Reporting Results of Internet E-Surveys (CHERRIES) [23] and Consolidated Criteria for Reporting Qualitative research (COREQ) guided the reporting of the data.

The research team comprised an honours’ physiotherapy student (BE), a masters physiotherapy student (RR), six physiotherapists (AC, LH, MP, HM, RR), and a registered nurse (AW). Several of the team (AC, LH, MP, HM, AW) are academics with experience in disability research and the use of qualitative and mixed methods research approaches. One of the broader team (ST-H) identifies as Māori, one of the team has lived experience of using MSE with a disabled family member, and a team member (HM) is a member of the Charitable Trust.

### 2.1. Objectives

Describe who used the MSE and why, using a retrospective survey, and then explore experiences of the room with a sub-group of room users and their support persons.

### 2.2. Recruitment and Procedures

#### 2.2.1. Quantitative Strand

E-Survey participants were recruited, via email, from a database of users who had used the MSE between 1 January 2019 and 31 March 2022. All room users who had accessed the MSE within these dates were eligible for inclusion. An invite email was sent to 1487 contacts from the MSE database, with a one-page study outline, information sheet, and a link to the anonymous e-survey, delivered via Qualtrics (RR, MP, LH). A hardcopy of the survey was also available on request. No incentives were offered for completion of the survey.

#### 2.2.2. Qualitative Strand

We invited for an interview: (i) support persons of neuro-diverse children (*n* = 15) who provided contact details in the survey; and (ii) disabled adult participants, aged ≥18 years, and their support persons (*n* = 12), who were approached by the MSE team leader, indicated they were interested in the study, and had provided contact details. For those who agreed to an interview, the researchers (BE, AW, ST-H) organised a convenient time, date, and mode of interview (email, phone, or internet platform e.g., zoom). A one-page outline of the study attached to a detailed information sheet and consent form were provided either in a standard or easy read format. Written informed consent was sought from the participant or the support person who usually supported decision making of the participant, if the participant was unable to provide written consent themselves [24].

### 2.3. Data Collection

#### 2.3.1. Quantitative Strand

Using a non-randomised, voluntary multiple-choice and open-ended question Qualtrics survey, we collected: (i) an indication of whether the respondent was the MSE room user (participant) or completing the e-survey on behalf of a room user (support person); (ii) participant demographics (age, gender, ethnicity, region where they resided, who (if anyone) accompanied the room user to the MSE, mode of transport, and frequency and length of SCMSE room use); (iii) barriers to access; and iv) reported participant disability via the Washington Group Short Set on Functioning (WG-SS), an internationally validated brief tool designed to conceptualise disability and for use in large population surveys [25]. This tool includes a set of six questions (4-point Likert scales) about functional domains of seeing, hearing, walking, cognition, self-care, and communication. These domains are most frequently found to measure what an individual cannot undertake within society’s disabling environment [25]. We presumed that as children would not be filling in the survey, use of the adult version of the WG-SS would be appropriate [25]. A back button was available in the e-survey for participants/support person(s) to review and change their answers. Adaptive questioning was used; therefore, the total number of items ranged between 14 and 21 items.

Prior to use, the e-survey was externally peer reviewed by members of the MSE staff with feedback provided regarding wording of the questions [26]. Informed consent was gained as part of the e-survey. For those who could not consent independently, assent was gained via the process of supported decision making from support person(s) (such as parents, guardians, relatives, or carers). Unique identity was determined using the matched email address. When an organisation email address existed rather than an individual email (e.g., when participants lived in a shared residential care facility), the organisation received the email and were asked to assist the participants in accessing the survey for completion. For this reason, the IP address of a client computer was not used to identify potential duplicates. All survey data were stored on an organisational, password protected database.

#### 2.3.2. Qualitative Strand

We developed a semi-structured interview guide with open-ended questions and prompts in key areas based on contextual understanding and previous research to capture data that corresponded to the research objectives (see Table 1) and refined it in consultation with the MSE staff. The interview guide provided a framework, although the wording, sequence, and follow-up questions were not intended to be fixed and could vary depending on what the participants or support person/s chose to discuss and how the interview evolved. We chose this approach to facilitate in-depth exploration of the participants or support person/s’ perceptions of the adult or child users’ experience of using the MSE. Questions regarding demographic information (age, gender, ethnicity) were also included. Interviews took place in person, via zoom, and via email, and were undertaken by four members of the team (BE, AC, AW, ST-H). Interviews were audio-recorded with permission and assent from participants/support person/s.

### 2.4. Data Analysis

#### 2.4.1. Quantitative Strand

Data were extracted to an excel spreadsheet, cleaned, and screened by RR. IP addresses were used to ascertain unique responses. The data were then coded, for example, the questionnaire completer was coded as the “participant”, “support person” or “parent/caregiver” (See Supplementary Material Table S1 Multisensory Room E-survey Codebook). Where a question was declined to be answered, or no answer was given, this was treated as missing data and no data were imputed. The data sets were exported into SPSS Statistics (IBM Version 28.0.1.0 (142)). Demographic data were analysed descriptively.

#### 2.4.2. Qualitative Strand

Interviews were transcribed verbatim, checked against the interview, and participant numbers were assigned to de-identify the participants to preserve anonymity (e.g., Interview 1 with adult participant and support person, AP1, and interview 1 with child participant and support person, CP1). We analysed the data using an Interpretive Descriptive (ID) approach, which has varying techniques for analysis [27,28]. Three members of the team (BE, HM, AC) used the research objectives to guide the initial deductive data analysis [29]. Each data set was read for familiarity and initial codes were assigned. Coding was reviewed by a further member of the team (MP), performing a consistency check to enhance the trustworthiness and clarity of key categories. Participant statements within each category were then inductively grouped using a manual scissor and sort technique and connected into thematic patterns of reoccurring ideas [27,28]. Summary categories were created, linked with the most important aims of the research [30]. Overall themes and subthemes were developed using an iterative reasoning process [27,28] and were agreed by the whole team. Meaningful quotes were then identified to conceptualise the findings and show consistency with the data. In the ID approach, the researchers’ personal belief systems and experiences cannot be separated from the research process and thus influence the interpretation of the data [28]. Data saturation was not required, as ID recognises that there is no limit to the variation of experience and that the data collected provides a nuanced understanding of a diversity of perspectives [31]. We did not engage in member checking as the process is unlikely to enhance the quality of the findings [32].

## 3. Results

Overall, 131 participants responded to the e-survey, representing a response rate of 8.8%. However, 27 respondents did not complete any questions after the consent question, and these data sets were removed, leaving 104 data sets for analysis. Of these 104 data sets, 18 (17.3%) respondents did not complete the survey in entirety. Not all the questions were answered by each participant. The e-survey was open for six weeks during June/July 2022 and took 15–20 min to complete.

The demographics of the survey and interview participants are provided in Table 2 and Table 3. These results show that the respondents were predominantly parents of young children with a disability. Of the 61 (80%) survey respondents who reported a disability, 42 (68%) reported having three or more WG-SS domains of disability. Among the respondents that reported their gender and ethnicity, most of the room users were male and New Zealand European.

Fourteen semi-structured interviews were undertaken. Seven adult support persons and one disabled adult participant were interviewed. The seven disabled adult participants gave assent for their support persons to share or vocalise their experience on their behalf and were present during the interviews.

Six adults discussed the MSE experience of eight children. Only one child room user was present during the interviews and briefly interacted with the interviewer. One child support person attended the MSE with three child participants. The interviews took between 20–50 min, and were conducted face-to-face (n = 6), via zoom (n = 6), email (n = 1), and telephone (n = 1).

Most of the child room users were male; conversely, most of the adult users were female. The types of limitations, as per the WSS-GS, of the room users included: Seeing (n = 2), Hearing (n = 1), Walking (n = 6), Concentration (n = 10), Self-care (n = 14), and Communication (n = 9).

Table 4 shows that the room users mostly came to the MSE by car and visited once a year or less. The most frequently stated barriers to access were the MSE booking system and the upstairs location of the room.

Analysis of the qualitative data (interview data and open-ended responses from the survey data) led to four themes: (i) Self-determination; (ii) Enhancing wellbeing opportunities; (iii) the MSE itself; and (iv) Accessibility. These themes represented the adult and child participants and their support persons’ perspectives about their experiences of using the MSE, and their perceived experiential barriers to or facilitators for using the MSE. The themes are described below, illustrated with participant quotes.

### 3.1. Theme 1: Self-Determination

Choice and control, individualisation, independence, and safety were all salient aspects of personal self-determination emphasised by the support persons and participants as facilitators of their MSE experience. The adult and child support persons acknowledged that the opportunity to exercise individual choice and control over the environment supported and promoted the independence of the participants and that this was an integral aspect of the MSE. “*It’s really good, he knows that if he goes in that space, he can choose what he wants, where to start or where to go, or what to do from [whichever] activity (AP3).”* For support persons of child participants, attending the MSE facilitated a time for reflection on how sensory experiences from the MSE might be adapted and incorporated into the home environment to build the participants’ self-determination. The MSE was also considered a safe space to take child participants with compromised immune systems. “*She can choose what she wishes to interact with because we know the MSE is sanitised after [every] use. So, we know she can use the equipment safely (CP3)*.” Several support persons and one adult participant stated that having the enclosed space to use provided a comfortable and safe community space that they could explore on their own.

*It’s good to have something safe I can get out of the house and go to. Sometimes the world seems pretty scary. Lots of noise, people and sights. Everything moving fast and [I don’t] understand [it]. At the MSE I feel safe. I really enjoy being there … as I approach, I was so excited. I felt like I was going home to a safe place I love to be*.

(AP8)

### 3.2. Theme 2: Enhancing Wellbeing Opportunities

The support persons and adult participants shared how the MSE created opportunities for social connection with others, influenced the room user’s behaviour and mood, and provided respite and a space to extend therapy. The MSE provided a recreational outing in the community, giving the participant an alternative activity in which to participate. The room created an opportunity for interaction, communication, and social connection with different people outside of the home unit, namely with the MSE staff. *“I love chatting to [staff (x)] during my session. She’s so friendly and nice. It’s great to have someone to talk to and I really enjoy that.” (AP8).* The perspectives on the effects of the MSE on the individual’s behaviour varied. Several of the support persons had observed that the MSE significantly contributed to improving the adult participants’ mood.

*When you tell him that you’re going to MSE, he starts clapping and laughing. Once he’s been, he is jumping and clapping. You can tell with his moods that he enjoys it …. I feel that he gets quite a lot of happiness and pleasure out of it.*

(AP1)

Multiple support persons believed that sensory stimulation helped to calm and settle the participants, improving their behaviour and reducing their aggression levels. “*After taking him to the sensory room it has a good effect on him. Usually, he will be calm the rest of the afternoon” (AP3).* Conversely, one support person stated that the MSE had no effect on the participant’s behaviour, instead the purpose was to engage purposefully with specific equipment. Several of the child support persons identified that access to the MSE enabled respite (time away from caring), in a safe space for the child.

*It helps [daughter] regulate and it’s become part of [her and our family] routine. ... It’s always a lovely time [staff member] knows all the ins and outs of [daughter’s name]. I can plonk down in the massage chair and it’s a lovely time for both of us*.

(CP5)

A different support person of a child participant used the time to extend speech therapy because the child was more receptive. “*When [the child’s] in a space where [the child] is having a great sensory experience, it’s a good time for me to [incorporate] some speech therapy techniques (CP1)*”.

### 3.3. Theme 3: Engagement in the MSE

Environmental factors, such as the room design, the role of the MSE staff, and implicit room rules, either facilitated or created challenges in the engagement in the MSE for both adult and child participants. The support persons stated that the physical room design was accessible and usable, and the equipment was in good condition. However, they recognised that the room design did not necessarily meet all of the needs and abilities of the adult users (e.g., space, colour) and suggested that the activities were catered more towards child users. The adult users appreciated a consistent, predictable, and familiar environment, with no unanticipated changes. “*He really has that routine [when he comes]. For [adult participant] that set [routine] ... going around and [equipment] is in the same place [is what he likes]” (AP3).* Changing activities and variability within activities was, however, acknowledged as good for stimulating interest and keeping the room entertaining. Differing perspectives on the frequency of change were noted. Introducing new activities slowly, while maintaining a degree of familiarity, was thought to assist those who found change difficult. One adult participant believed the current level of change provided a good balance between consistency and variability.

*I think it’s about right. … I get quite excited at my first session of a new theme. I enjoy looking at all the cool new things. For me, if it changed more often, I’d get too anxious, and if it changed less often, I wouldn’t have the excitement of looking at all the new things. So, it’s the right balance for me.*

(AP8)

Conversely, the child support persons believed that change and variation in the equipment were not frequent enough to keep the environment novel and fun for the children to explore regularly.

*Changing up the look of the room or changing some equipment [would be good]. Have an indoor swing or a slide for a child to interact with and to make the room look different when entering” (CP3) or having some things that are vestibular stimulating, some movement-oriented opportunities such as a tower to climb, a hammock to sit in.*

(CP1)

Additionally, the only Māori child support person who participated in an interview identified that the MSE did not reflect the Māori worldview, in line with her experience in other aspects of community life. *“[The MSE is not] reflective of our identity but I just find that normal ... so sad” (CP6).* This support person suggested that small changes, such as incorporating spoken and written Māori language and the use of culturally meaningful artifacts, would make the space more welcoming for Māori users.

*There’s lots of opportunities [that would enable Māori to feel more welcome and that would encourage the participant to want to tell others of this MSE opportunity]. I* *t would be good if someone on the staff was Māori. Someone to connect with them (the users), to greet them, to say kia ora. [It would also be good] to have te reo words around. It would be so cool if there were more Māori things [in the room]* *like taniwha, poi, and waiata and Māori themes like Matariki in the displays, pumice and paua in the ocean display, and waka on the wall.*

(CP6)

Many of the adult support persons appreciated the staff’s suggestions of different ways participants could interact with the room as this helped to keep each session interesting and engaging. “*She shows us the new things” (AP1).* One adult participant valued the MSE staff showing them new changes in the room, as this facilitated refamiliarisation with the MSE, and then they had independence to explore the room by themselves. “*When the theme’s changed, sometimes [staff name] shows me around [because] when I step in my brain goes ‘ahhh it’s different!’ Once I’m comfortable again, I enjoy exploring on my own” (AP8).* Some support persons/participants had, over time, developed meaningful relationships with the MSE staff. They valued the MSE staff making the effort to get to know participants and having an awareness of equipment preferences. A child support person noted that it was about familiarity (same MSE staff there every session) and that it was this staff member knowing the participant’s needs that was important to them. “*[The MSE staff member] gets out additional equipment that suits my daughter” (CP3)*. An adult support work also echoed the importance of the staff remembering what participants liked to engage with. They observed that “*it was a really [good thing] when the [staff member] remembered that [name] liked those things and actually took the time to get that [equipment] out for [name]. [Name] really appreciated that” (AP2).* However, one support person stated that while they appreciated the support provided by the MSE staff, they felt it was more beneficial for the participant to provide direction on what was to be engaged with, to facilitate the room user to maintain independence, make autonomous decisions, and thus feel like they can explore freely. In other words, they felt it was important for the MSE staff to recognise that users may require or wish for different levels of attention and involvement and that the MSE may require individualising to/for the user needs each visit.

*Whoever’s leading the session always says “it’s time to finish [the session]. Come and choose somewhere to lie down.” … And that’s quite lovely, but sometimes you just get into engaging with an activity [with the participant] and it’s time to come and lie down. It just seems like a bit of a waste of time … there doesn’t sort of seem to be an option to say, “No we don’t want to do that now … [participant] might like a bit more time [doing what] she really wants to have more turns at and loves”.*

(AP2)

Several support persons suggested that it would be helpful to know the MSE ‘rules and procedures’ as they found it difficult to determine whether there were any expectations of the “room user” when using the MSE, for example, putting the equipment back where they found it. *“We don’t have a lot of interaction with the staff. I guess that’s a little bit hard to know what their role is, … to know what we are allowed to do and what we’re not. You know, do we have to put equipment back, [or] is the MSE staff member going to put it back?” (AP2)*. It was suggested that clarification regarding how they are allowed to use the room, what is expected of them, and what they could expect from the staff would be beneficial.

### 3.4. Theme 4: Accessibility

The participants predominantly described external environmental barriers (such as the MSE being upstairs), rather than internal barriers (i.e., lack of time) to access. This is similar to the findings from the survey (Table 4). Most of the concerns from the surveys and from the qualitative data were related to physical access to the MSE once having entered the council facility, booking, and paying for sessions, and limited accessible information about the MSE. *“It’s a shame it’s not easier to [get to, book and pay], we would use it regularly” (CP1, CP6)*. The survey respondents indicated that physical access issues included the *“elevator is too small for prams” (R100)*, *“it’s a bit of a pain [the MSE] being upstairs” (R103)* and *“[participant] needs assistance to walk up the stairs to the sensory room” (R12)*. Within the interviews, the support persons provided more information about physical access to the building and the challenges this presented. Entering through the main entrance of the facility was described as fatiguing and stressful. The location of the MSE within the overall facility reduced its accessibility, especially for those with reduced mobility. Being in the back corner of the building on the second floor, as well as having limited parking available close by, increased the distance and time required to access the room. Additionally, navigating crowds of people and other distractions (e.g., the café, the merchandise stands, and shop) created challenges for the support persons and participants “*Yeah … you got the cafe, the shop, the vending machines, the counters, you’ve got potentially about 20 odd people between there and the stairs, and so it’s too much [stimulation] for [participant]” (AP7)*. The few interviewees who knew about a back entrance to the facility believed it provided a more straightforward path of access, reduced the distance to travel, and minimised the number of distractions and challenges enroute. *“I’m so happy the back door is finally open now so I can just come in and avoid all the people” (AP8).*

Both the survey respondents and support persons and participants described the telephone booking system as frustrating. It was hard to obtain a booking, they could not book online, and finding suitable times to use the MSE was difficult. Survey respondents stated the *“Council booking system! [There are] never any spaces around the times that suit” (R4), “[I’m] having trouble…booking the room over the phone” (R34), “why can’t we book online” (R70),* and *“it is so popular, getting a time that suits is difficult” (R101).* The interviewed support persons and participants discussed how having a fixed, regular session booked created a routine activity that the participants could plan for and look forward to. However, if the participant’s needs or plans changed, sometimes the individual missed sessions. Having the ability to book a regular session for a shorter period, such as for a school term instead of yearly, along with implementing a cancellation policy, was suggested to reduce the challenges and frustrations related to the booking system.

*With the kind of participant [we bring to the MSE], things change [at short notice] with them you know. If you could just book for the school terms, … [and] there was the facility to change or move the booking to times that would suit participants better would be good. For now, we’re stuck with a booking for the whole year*.

(AP5, 6)

One adult participant appreciated being able to book sessions via email, as this reduced the requirement for in-person communication. The fixed length of the sessions was also raised as something that could be more flexible. Some of the support persons reported that the participants did not use the full allocated 30 min, while others stated that they needed longer sessions to allow for better engagement. The ability to attend group sessions was also raised as a potential way to facilitate engagement with others while in the room *“if there was a time on Saturday afternoon, when you could just go (without a booking) and there were others using the room too, then we could actually do that [because it would be useful social learning for child]” (CP1)*. However, the ability to book individual sessions was valued by the support persons and participants as it guaranteed the individual time, with no crowds, interruptions, or distractions.

*It’s just that things take longer for us. Sometimes it takes maybe 15 min to warm up to being in a new space and explore until we find something that we like. Then [there’s only] five minutes to do that [activity] and it doesn’t seem like enough time. Probably 45 min would be a better time frame [for a session].*

(AP2)

CP3, a child participant support person, described the medications and respiratory equipment that was needed for the child to be taken on this sort of outing. They identified that the considerable effort to pack all this gear into the car for a 30-min experience seemed almost overwhelming, and that a longer session would feel much more ‘worthwhile’.

Explicit information about payment options (i.e., the pre-paid card option) for room users would decrease the support persons’ frustration and enable them and the participants to avoid the foyer and enter the building through the back door.

*I used to pay for the whole year. But then there’d be different staff on at the front desk and they didn’t know us. I’d say, “We’ve paid for the whole year” and they’d say, “No you can’t do that, blah blah” and you’d be there for 10 min trying to explain to them. We’ve got a card now and we just swipe it … I just wish we had known about the pre-paid card three years ago when we started all this hoo-hah.*

(AP3)

An option to top up the prepaid cards online would improve the payment system, allowing the support persons and participants to independently manage the payment and reduce the challenge of in-person communication at the facility. In addition, many of the support persons told us that the information available about the MSE did not sufficiently inform the support persons and participants of the room purpose, location, and how to effectively engage with it or support someone exploring it. A lack of explicit advertising of the room was also noted. However, one participant did appreciate the online photos of the MSE, which provided some information on what to expect: *“there were pictures of the room itself online so I knew what it would look like inside”* (AP8). Multiple support persons and participants suggested that providing a video tour or a map of directions, with instructions on how to access the room once inside the recreational facility would reduce one of the accessibility challenges for people unfamiliar with how to get to the MSE itself.

*I was really anxious [before] going the first time, and a big part of that was not knowing where to go when I got to the recreational facility, or how to check in. … I would have loved a video tour on the website that shows going in the main entrance, walking up to the counter and what to say, and then where to walk from there. I couldn’t find a map of the recreational facility online, so if not a video, a map would help, and step by step instructions.*

(AP8)

## 4. Discussion

Multisensory environments (MSE) are an emerging concept and there is limited evidence exploring users’ experiences of community MSEs, and less about disabled people experiences of such spaces. This study contributes to that knowledge gap. The exploration of who used the MSE suggested that while the response rate for the e-survey was low, the respondents were predominantly young and NZ European, with the most frequent reported disabilities being in the areas of self-care, communication, and concentration. Māori disabled people (tāngata whaikaha) were underrepresented as users in the e-survey, at 13%, compared to the age-adjusted population data. Internationally and nationally, disabled people experience higher health inequities and unmet health needs when compared to non-disabled people [33,34], with Indigenous peoples experiencing higher rates of health inequities [35]. In the NZ 2013 Disability Survey, 24% of people identified as disabled [36], with disproportionately higher rates for tāngata whaikaha (32%) when adjusted for age [36]. Findings from the 2021 NZ Health Survey highlighted that 7.6% of disabled people had reduced access to general practitioner (GP) care due to transport compared to 1.9% of the non-disabled population, and 16% of disabled people struggled due to the cost of GP care compared to 9.7% of the non-disabled population [33]. Subsidised community run spaces, such as the MSE in this study, could provide disabled people an enjoyable destination for improving their long-term health, social connection, and well-being [37].

The participant experience of the MSE in this study suggested that the development of their capacity for self-determination [38] was supported. The freedom to make one’s own choices and control your environment are a basic right that should be respected, regardless of the individual’s abilities and support needs [13,39,40]. Choice and control, in turn, support overall well-being and behaviour, and affect, an individual’s sense of identity [39]. The opportunity for choice promotes and enables self-determination, which is an important contributor to developing a sense of personal empowerment and enhancing quality of life for disabled people [39]. The idea of user control is a key concept of MSEs that is consistently discussed in the wider literature, and user control influences the overall MSE experience for disabled people [41,42,43,44]. Choice, agency, and control over environments motivate individuals with profound and multiple disabilities to explore environments freely [5].

Creating a space that is welcoming for all and that is considered culturally safe by Indigenous peoples (in NZ, that is Māori) is suggested to assist in addressing health disparities [45]. In this study, the MSE space was identified as unwelcoming for Māori users and potentially others who struggled with the booking and payment systems, as well as accessing the room itself. The development of systems and ways to make the space more welcoming for Māori and other groups, improve access, and for customising the MSE for room user preferences (i.e., session length, individual and group sessions, facilitation of session, tolerance for change, equipment likes and dislikes), together with room users, can only enhance user experience. Practices that privilege co-design (such as He Pikinga Waiora (Enhancing Wellbeing) Implementation Framework [46]) with people who use the MSE would enhance and extend the relationship and connectedness, which are important for health and wellbeing [37]. Furthermore, the facilitation of further “joint problem solving” [47] discussions around what is important to develop for users and what to include in the room would make it more welcoming for all.

The accessibility of the MSE for disabled people was highlighted as the biggest issue in this study. Accessibility, as a concept, remains difficult to define as the idea has been shaped by multiple discourses and perspectives [48]. Accessibility is now understood to not only encompass one’s proximity to something, but also to include, for example, how information is displayed, the availability of amenities and how these are booked, arrived at, and used, and of the place or space being cultural inviting [20]. Essentially, if community spaces such as MSEs were truly ‘accessible’, then, as welcoming spaces for *all*, they would facilitate self-determination (choice and control) about how and when to use the space [20], and provide opportunity for active, self-directed participation in the chosen space and its community [16].

### 4.1. Strengths and Limitations

This study adds to the very limited literature available on disabled persons’ experiences of using an MSE. A great strength of the study was that the research question was community driven, rather than researcher derived. The study design facilitated an increased understanding of disabled adults and children’s experiences of using a community MSE. The survey only went to previous MSE users, thereby limiting the understanding of barriers to access for people who have not used the room. Furthermore, the council were unable to provide us a full data set of all registered service users, citing privacy reasons; thus, the representativeness of the survey respondents is unknown. Expansion to a wider audience could be completed in conjunction with a broader range of local disabled people’s organisations. Definitions of access to healthcare exist, yet measurement of this concept is challenging [49]. The use of binary (yes/no) questions did not explore the concept in enough depth, limiting the reliability of the study findings. However, using other methods to address the research question provided the opportunity to talk with the participants about their experiences of using the room. Interviewing the support persons and participants together allowed those with limited opportunity and ability to express their experiences and preferences to be represented in the research. The use of supported decision-making enabled the equitable participation of people who were non-verbal or unable to provide written consent to participate in this study.

### 4.2. Recommendations

Overall, the participants believed that the MSE experience could be enhanced by addressing the access challenges and broadening the scope of equipment to improve the usability and make it a more inclusive environment for all ages. Exploring with Māori and Pacific Peoples and other communities about their views and needs would contribute to improving the accessibility and usability of the community space. In addition, the geographical location, with consideration of affordable transport links, where the room is situated in the facility, flexible room booking and opening hours, and the cost of each session requires careful consideration with end-users.

## 5. Conclusions

The exploration of disabled users’ experiences of a community-based MSE and the inclusion of their voice within the research process showed that the MSE supported self-determination and enhanced wellbeing opportunities. The participants appreciated the opportunity to use this community facility. However, the MSE experience could be enhanced by addressing the access challenges and broadening the scope of equipment to improve the usability and make it a more inclusive environment for all. We suggest that there may be a potential benefit in having other similar facilities in the community and/or to explore the use of mobile sensory spaces, which could offer meaningful sensory activities to those who are unable to access static community MSEs.

## Figures and Tables

**Table 1:** Semi-structured interview guide.

Topics	Interview Guide
Demographics	Age, gender, ethnicity/iwi
General experiences of the multisensory room	Can you share your thoughts along with some examples of your experiences of using the multisensory room? Prompts: Reasons for using the room, benefits, or barriers, if you could change anything in the room what might it be and why?
Equipment	Could you share your thoughts about your equipment preferences? Prompts: What equipment do you enjoy using and why? Is there other equipment that you would like added or removed from the multisensory room? Please explain.
Accessibility	Talk me though what is involved for you (and your support persons) in getting ready and then getting to the multisensory room. Prompts: Transport, the path of travel from the building entrance to the multisensory room Talk me through what is involved for you (and your support persons) in the return journey, from the multisensory room to home. Can you share your experiences and some examples about the accessibility of information about the multisensory room. Prompts: Can you tell us about how you found out about the multisensory room (i.e., who referred you and why?). What information was available (e.g., online, brochures)? Did the information available meet your needs (i.e., was there enough information or too much)? Where and how did you go about finding further information if you needed to? Is there anything that could be done differently to enhance the information about the multisensory room?
Support needs	Who accompanies them to the room. Understanding the impairments, they experience/sensory systems affected

**Table 2:** Demographics of e-survey respondents.

E-Survey Respondents			Total Frequency n = 104 (%)
Type of survey completer			N = 101 (96)
		Parents	74 (73)
		MSE room user	15 (15)
		Support person	12 (12)
Room user			
	Gender		N = 29 (27.9)
		F	8 (27.6)
		M	21 (72.4)
	Age (years)		N = 96 (92.3)
		<4	45 (46.9)
		5–21	32 (33.3)
		>21	19 (19.8)
	Ethnicity		N = 104
		multiple responses totalling 107 (102)	
		Māori	14 (13.1)
		New Zealand European	81 (75.7)
		Pacific People	2 (1.8)
		Other	10 (9.3)
	Disabled person		N = 76 (73.0)
		Yes	61 (80)
		No	15 (20)
	Types of limitations (WG-SS)		N= 76
		multiple responses totalling 211 (200)	
		Seeing	11(5.2)
		Hearing	6 (2.8)
		Walking	32 (15.2)
		Concentration	47 (22.3)
		Self-care	58 (27.5)
		Communication	57 (27.0)

Note. MSE: Multisensory environment; WG-SS: Washington Group-Short Set.

**Table 3:** Demographics of interview participants.

Interview Participants N = 14			MSE Users N = 16
Disabled adult room users			
	Gender	Male	n = 3
		Female	n = 5
	Age	20–70 years old	
	Ethnicity	Māori	n = 1
		New Zealand European	n = 6
		Other	n = 1
	Accompanied by support worker		n = 7
Child room users			
	Gender	Male	n = 5
		Female	n = 3
	Age	1–11 years old	
	Ethnicity	Māori	n = 1
		New Zealand European	n = 6
		Other	n = 1
	Accompanied by support person/s		n = 6

**Table 4:** Access to the multisensory room.

E-Survey Respondents		Total n = 104 (%)
Frequency of room use per year		N = 104 (100)
	Every 2 weeks	8 (7.6)
	Monthly	20 (19.2)
	2 to 4 times a year	25 (24.0)
	Once a year or less	51 (49.0)
Transport to the MSE		N = 85 (80.9)
	Bike	1 (1.2)
	Bus	3 (3.5)
	Car	72 (84.7)
	Mini Van	5 (5.9)
	Walk	4 (4.7)
Barriers to MSE access		N = 39 with multiple responses 43 (110)
	Booking system	11
	Distance to MSE	4
	Individual time constraints	6
	Location of front desk	3
	MSE too overwhelming	2
	Staff shortages	5
	Upstairs location	8
	Other	4

Note. MSE: Multisensory environment.

## Data Availability

The data are not publicly available because participants have not consented to the sharing of their data for secondary analysis.

## Supplementary Materials

The following supporting information can be downloaded at: https://www.mdpi.com/article/10.3390/ijerph20196805/s1, Table S1: E-survey codebook.
