# Supplementary material for: Disabled People or Their Support Persons’ Perceptions of a Community Based Multi-Sensory Environment (MSE): A Mixed-Method Study
Source: Int J Environ Res Public Health. 2023 Sep 22;20(19):6805. doi: 10.3390/ijerph20196805 (PMC10572127; doi:10.3390/ijerph20196805)
Supplement: Supplementary file 1 [file ijerph-20-06805-s001.zip › ijerph-2393218-supplementary.pdf]

Table S1 – Multisensory Room E-survey Codebook

Question #	Variable	SPSS variable name	Coding instructions
	Identification number	ID	Number assigned to each questionnaire
Q1	Questionnaire completer	Completer	1 = Room user<br>2 = Support person<br>3 = Parent/Caregiver
Q2	Age	Age	Age in years, recoded to NZ Ministry of Education funding models of four categorical groups.<br>1 = 0-4,<br>2 = 5-21,<br>3 = 22-64<br>4 = 65 plus<br>For secondary analysis of relationships between variables age recoded to<br>1 = Under 21<br>2 = Over 21
Q3	Ethnicity	Ethnicity	Used NZ Statistics Department standard ethnicity question.<br>1=New Zealand European<br>2=Māori: Please give the name(s) and region(s) of your iwi (tribe or tribes)<br>3=Samoan<br>4=Cook Island Māori<br>5=Tongan<br>6=Niuean<br>7=Chinese<br>8=Indian<br>9=Other such as Dutch, Japanese, Tokelauan. Please state:<br><br>Recoded as:<br>1=NZEuro<br>2=Māori<br>3=Other<br><br>A particular interest of the Trust was to find out how many Māori used the MSE, any person who identified as Māori were categorised as Māori and all other respondents were categorised as non-Māori.<br>For secondary analysis of relationships between variables, ethnicity recoded to<br>1 = Māori<br>2 = Non=Māori

Table S1 – Multisensory Room E-survey Codebook

Q4	Region	Location	1= Canterbury<br>2 = Other, South Island<br>3 = North Island
Q5	Who accompanies person	Companion	1 = No<br>2 = Yes
Q6	Transport	Transport	1= Bike<br>2 = Bus<br>3 = Car<br>4 = Mini Van<br>5 = Walk<br>6 = Other
Q7	Frequency of use	Frequency	1 = Every 1 -2 weeks<br>2 = Monthly<br>3 = 2-4 times a year<br>4 = Yearly<br>5 = Other<br><br>For secondary analysis of relationships between variables, frequency recoded to<br>1 = Monthly or more frequently<br>2 = 2 – 4 times year<br>3 = Yearly or less
Q8	Barriers to access	Accessibility	Reviewed for themes then coded
Q9	WG-SS	WG-SS	WG-SS recoded to:<br>1 = has disability (A lot of difficulty, Cannot do at all)<br>2 = no disability (No difficulty, Some difficulty)<br>With 'A lot' or 'Cannot do at all' in any type of limitation codes as Yes = Disabled
Q10, 14-15	Long-answer - factors influencing decision to attend, how to advertise MSE, other information to support MSE experience		Reviewed for themes
Q12	Accessibility barriers	Barriers	1=yes (if yes please explain)<br>Explanations provided reviewed for themes.<br>2=no

Table S1 – Multisensory Room E-survey Codebook

			Reviewed for themes then coded
